# Comparing self and informant perspectives on neuropsychiatric symptoms in older adults in a memory clinic

**DOI:** 10.1177/13872877251401620

**Published:** 2025-12-15

**Authors:** Monika Krejčí, Veronika Matušková, Toni T Saari, Hana Horáková, Šárka Borovská, Jan Laczó, Jakub Hort, Martin Vyhnálek

**Affiliations:** 1 Department of Psychology, Faculty of Arts, Charles University, Prague, Czechia; 2 Memory Clinic, Department of Neurology, Second Faculty of Medicine, Charles University and Motol University Hospital, Prague, Czechia; 3 Department of Clinical Psychology, Motol University Hospital, Prague, Czechia; 4 Institute for Molecular Medicine Finland (FIMM), HiLIFE, University of Helsinki, Helsinki, Finland

**Keywords:** Alzheimer's disease, mild behavioral impairment-checklist, mild cognitive impairment, neuropsychiatric symptoms, self-informant comparison, subjective cognitive decline

## Abstract

**Background:**

In individuals with cognitive deficit, neuropsychiatric symptoms (NPS) are typically assessed through informant-based reports. In contrast, affective symptoms in cognitively normal individuals are typically evaluated using self-report measures, leveraging their sufficient insight. Comparison of these assessments in previous studies indicated differences in their perspectives.

**Objective:**

We aimed to analyze differences between self- and informant-reported NPS using the Mild Behavioral Impairment-Checklist (MBI-C) and their relationship with cognitive deficit.

**Methods:**

The study included 127 participant-informant dyads from a memory clinic cohort, classified as subjective cognitive decline (SCD; N = 38) or mild cognitive impairment (MCI; N = 89) based on comprehensive neuropsychological assessment; MCI group was diagnosed according to core clinical criteria for MCI due to Alzheimer's disease. A control group consisted of 26 cognitively healthy individuals. Participants and their informants completed the MBI-C, evaluating five symptom domains.

**Results:**

Both self- and informant-rated MBI-C scores were higher in the SCD and MCI groups compared to controls. Informant ratings correlated more strongly with objective cognitive deficit, particularly in memory and executive function domains, whereas self-ratings showed only one association (higher apathy with worse attention). In MCI, informants reported more severe impulse dyscontrol symptoms. Correlations between self and informant ratings were weak to moderate.

**Conclusions:**

This study highlights discrepancies between self- and informant-rated NPS assessments, with informant ratings more strongly linked to cognitive deficits in older adults without dementia. Therefore, we suggest using both assessments to capture informants’ and patients’ subjective experiences and facilitate early identification of neurodegenerative diseases.

## Introduction

Neuropsychiatric symptoms (NPS), changes in mood, behavior, or personality in older individuals, can be part of normal aging but may also indicate early neurodegeneration including Alzheimer's disease (AD).^1^ These symptoms often emerge before dementia, i.e., in mild cognitive impairment (MCI) and subjective cognitive decline (SCD).^2^ New-onset NPS in these populations are associated with a higher risk of developing dementia in AD.^3^ Informant reports further suggest that NPS severity increases with advancing cognitive impairment.^4^

To raise awareness about NPS as early symptoms of pathological aging, a syndrome termed mild behavioral impairment (MBI) was introduced. MBI refers to persistent new-onset NPS in adults aged 50 + without dementia. These symptoms cannot be attributable to another current psychiatric disorder (e.g., major depression) and should cause at least mild functional impairment.^5^ MBI can be diagnosed concurrently with MCI but can also precede any objective cognitive deficit.^6^ Studies have indicated that MBI reflects neurodegenerative changes in the brain and that it can be present in any neurodegeneration including AD.^7–9^ To assess MBI, the Mild Behavioral Impairment Checklist (MBI-C) was developed.^10^

In dementia, NPS are typically evaluated by informants (a relative or other close person) due to patients’ reduced insight.^11^ In cognitively normal or mildly impaired individuals, NPS are often assessed via self–rated questionnaires.^12,13^ Given the subjective nature of symptom reporting through questionnaires, informants may differ from patients, potentially having significant implications. Self-informant rating discrepancies in cognitive complaints showed potential to predict incident cognitive decline in participants at risk of AD, which led to the formation of a specialized working group.^14^ Similar discrepancies appear in affective symptoms, with greater differences observed in more pronounced cognitive deficit.^15–17^ Such differences may stem from both patient- and informant-related factors: reduced patient insight due to memory or executive deficits and caregiver burden leading to overestimation of symptoms.^18–20^ However, most previous studies used different instruments for patients and informants, limiting the direct comparability of their ratings. One exception is a study using the Apathy Evaluation Scale, showing higher self-rated apathy in cognitively normal individuals, but lower in MCI patients at risk of AD, compared to external assessments.^16^

The MBI-C, suitable for both self and informant rating, enables direct comparison. Recently, patient administration has begun to be used more often.^21–23^ However, only a few studies have directly compared both perspectives.^24,25^ They concluded that both informants and participants with SCD and MCI most frequently reported affective dysregulation, impulse dyscontrol and decreased motivation. It is important to note that their ratings differed in severity and correlated only weakly.^24,25^ Notably, these studies involved community-dwelling older adults without comprehensive neuropsychological assessment. No study to date has examined MBI-C self-informant discrepancies in a clinical population.

Our study addresses this gap by examining self-informant differences in the MBI-C in a memory clinic sample of older adults without dementia. Across the cognitive continuum, we aim to describe and compare the severity of NPS reported by participants and informants and to investigate the self-informant agreement in NPS ratings and its relation to cognitive impairment. We hypothesize an increase in NPS severity across the cognitive continuum (lowest in controls, higher in SCD, highest in MCI, for both self and informant ratings). Furthermore, we expect only modest self-informant associations in NPS ratings, resulting in a discrepancy in their MBI-C scores. These self-informant differences will increase with cognitive impairment, particularly in memory and executive function.

## Methods

### Participants

The study included 127 participant-informant dyads referred to a memory clinic for cognitive complaints and subsequently enrolled in the Czech Brain Aging Study.^26^ Based on comprehensive neurological and neuropsychological examination, participants were classified as SCD or MCI. SCD (*n* = 38) was diagnosed using the SCD-Initiative Workgroup criteria and required subjective cognitive decline without objective impairment in the neuropsychological assessment based on age-, sex- and education-adjusted norms.^27^ MCI (*n* = 89) was diagnosed according to core clinical criteria for MCI due to AD, and included persons with subjective cognitive complaints, neuropsychologically confirmed objective impairment (>1.5 SD below norms on ≥2 tests within a domain) and preservation of functional independence.^28^ Both amnestic (*n* = 79) and non-amnestic (*n* = 10) MCI were included. All diagnoses were made by consensus involving cognitive neurologists and neuropsychologists.

Additionally, a control group of participant-informant dyads was included (*n* = 26). Controls were cognitively healthy volunteers (from the University of the Third Age or patient's close relatives) with no significant subjective cognitive complaints and normal performance on neuropsychological testing.

Exclusion criteria included a diagnosis of dementia and other neurological or psychiatric condition significantly affecting cognition or NPS (e.g., Parkinson's disease, frontotemporal lobar degeneration, traumatic brain injury, stroke, alcohol or substance abuse, or current/past major psychiatric disorder). Demographic characteristics of the participants are presented in Table 1.

**Table 1.** Demographic and neuropsychological characteristics of the participants.

Characteristics	CN (*n* = 26; *M* ± *SD*)	SCD (*n* = 38; *M* ± *SD*)	MCI (*n* = 89; *M* ± *SD*)	Group differences
Age^a^	70.5 ± 5.07	67.1 ± 8.30	73.8 ± 7.28	CN < MCI*; SCD < MCI***
Female, *n* (%)^b^	24 (92.31)	31 (81.58)	44 (49.44)	CN > MCI***; SCD > MCI***
Education^a^	16.5 ± 1.90	15.9 ± 2.81	15.7 ± 3.16	–
MMSE, score^a^	29.62 ± 0.64	29.21 ± 1.14	27.01 ± 2.08	CN > MCI***; SCD > MCI***
WAIS-III Digit Span, score^a^	16.85 ± 4.35	16.13 ± 2.78	14.04 ± 3.06	CN > MCI*; SCD > MCI***
Digit Symbol, score^a^	43.96 ± 11.39	42.87 ± 7.74	31.15 ± 10.72	CN > MCI***; SCD > MCI***
TMT A, time to completion (s) ^a^	40.52 ± 11.22	41.00 ± 12.38	59.72 ± 28.52	CN < MCI***; SCD < MCI***
TMT B, time to completion (s) ^a^	82.13 ± 22.46	83.03 ± 16.46	176.21 ± 82.65	CN < MCI***; SCD < MCI***
P-VF, score^a^	49.65 ± 10.24	54.97 ± 11.76	38.89 ± 11.32	CN > MCI***; SCD > MCI***
C-VF animals, score^a^	26.96 ± 5.88	27.03 ± 5.43	18.49 ± 5.63	CN > MCI***; SCD > MCI***
BNT-30, mistakes after a semantic cue^a^	2.42 ± 2.00	1.45 ± 1.37	4.61 ± 3.80	CN < MCI***; SCD < MCI***
LM delayed recall, score^a^	17.96 ± 3.68	17.03 ± 3.28	8.22 ± 5.62	CN > MCI***; SCD > MCI***
RAVLT 1–5, score^a^	56.23 ± 7.86	56.58 ± 8.10	37.72 ± 8.95	CN > MCI***; SCD > MCI***
RAVLT delayed recall, score^a^	11.77 ± 2.36	11.71 ± 2.19	4.65 ± 3.23	CN > MCI***; SCD > MCI***
ROCF copy, score^a^	30.96 ± 3.09	31.00 ± 2.60	26.56 ± 6.16	CN > MCI***; SCD > MCI***
ROCF recall, score^a^	19.58 ± 6.53	18.83 ± 4.26	9.92 ± 6.12	CN > MCI***; SCD > MCI***

CN: cognitively normal; MCI: mild cognitive impairment; SCD: subjective cognitive decline; MMSE: Mini-Mental State Examination; TMT: Trial Making Test; P-VF: phonemic verbal fluency – letters N, K, and P; C-VF: category verbal fluency; BNT-30: Boston Naming Test – 30-item version; LM: logical memory; RAVLT: Rey Auditory Verbal Learning Test; ROCF: Rey–Osterrieth complex figure.

^a^ ANOVA with Games-Howell post hoc test, ^b^chi-square.

*p < 0.05; **p < 0.01; ***p < 0.001.

All participants provided written informed consent according to the Declaration of Helsinki and the study was approved by the Ethics Committee of Motol University Hospital.

### Neuropsychological assessment

The neuropsychological battery included the Mini-Mental State Examination (MMSE) as a screening of global cognition and the following tests covering five cognitive domains^28,29^: (1) memory: Rey Auditory Verbal Learning Test (RAVLT),^30^ Logical Memory from the Wechsler Memory Scale-III (LM),^29^ and 3-min recall of the Rey-Osterrieth Complex Figure Test (ROCFT recall)^31^; (2) executive function: Trail Making Test B (TMT B)^29^ and phonemic verbal fluency – letters N, K, P (P-VF)^32^; (3) language: 30-item Boston Naming Test (BNT) and category verbal fluency-animals (C-VF)^29^; (4) attention and working memory: Trail Making Test A (TMT A) and Digit Span Forward and Backward (DS) from the Wechsler Adult Intelligence Scale-III^29^; and (5) visuospatial function: Rey-Osterrieth Complex Figure Test (ROCFT copy).^31^

### Neuropsychiatric assessment

The Czech version of the MBI-C was completed by both participants and their informants (partner, descendant, or another relative).^33^ The MBI-C is a 34-item questionnaire designed to assess NPS before the onset of dementia.^10^ It covers five domains aligned with MBI diagnostic criteria: decreased motivation (6 items), affective dysregulation (6), impulse dyscontrol (12), social inappropriateness (5), and abnormal perception and thought content (5).^5^ Each item is rated for presence (yes/no) and severity (1-mild, 2-moderate, 3-severe). The symptoms should represent a change from the person's usual behavior and persist over the last 6 months. Total and domain scores are calculated by summing severity ratings (e.g., total score is 0–102). Participants with ≥4 missing MBI-C items were excluded; if ≤3 items were missing, both total and domain scores were calculated without these items. In total, 41 items (0.4%) of the 10 404 patient and informant items were omitted. No single MBI-C item was commonly omitted; missing responses were distributed across items.

### Statistical analyses

Group differences in demographic and neuropsychological variables were assessed using ANOVA with Games–Howell post hoc tests, or chi-square tests for categorical data. Because MBI-C data distribution was skewed, nonparametric tests were used: Kruskal–Wallis tests (with Dwass-Steel-Critchlow-Fligner post hoc comparisons) to compare MBI-C scores among diagnostic groups, and Wilcoxon signed-rank test for paired data to compare informant- and self-ratings within each diagnostic group, visualized with ggplot2.^34^ Associations between informant- and self-rated MBI-C scores were analyzed using Spearman correlations and these correlations were compared between diagnostic groups using bootstrapping.

Associations between MBI-C scores and cognitive domains (memory, attention and working memory, executive functions, language, visuospatial functioning and global cognition) were examined using linear regression analysis. Cognitive domains were composite *z*-scores calculated as the averages of *z*-scores of administrated neuropsychological tests for each cognitive domain. Scores for TMT A, TMT B and BNT were reversed before being transformed into *z*-scores. Since it is plausible that informant and self-rated NPS could have both separate and combined associations with cognition and that these could differ by cognitive domain, multiple sets of analyses were conducted. To examine the effect of larger variance explained in cognition by using MBI-C informant and patient scores separately rather than informant-minus-patient difference scores, two competing regression analyses were run for each MBI domain predicting each cognitive domain. In the first, informant-minus-patient difference score was used, and in the second, the informant and patient scores were entered separately.

Only SCD and MCI participants data were included in the regression analyses, because most MBI-C scores in CN were zero. Data from SCD and MCI participants were combined to increase statistical power. MBI-C variables were log-transformed after adding 1 to MBI-C scores (see Supplemental Figures 1 and 2 for distributions before and after transformation). *p-*values were adjusted following Holm, considering a set of cognitive domains predicted by a single MBI domain as a family.^35^ In analyses with age, sex and education as covariates, only the *p-*values of MBI-C variables were adjusted since our primary focus was on MBI-C, not on the well-established associations of demographic factors with cognitive performance.

The assumptions of linear regression were checked with residual plots, Q-Q plots and checking the variance inflation factors. Most models met the assumptions of linear regression, but some models, particularly those with social inappropriateness or abnormal thought and perception content as predictors had deviations from these assumptions. On the whole, these deviations were not considered sufficient to alter the analytic strategy that aimed to examine associations of all MBI domains with multiple cognitive domains.

Analyses were run in and R 4.3.2, with *p* < 0.05 considered significant.^36^ According to a priori determined sample size using G*power 3.1.9.7, 27 dyads per diagnostic group was required to detect medium-sized effect in MBI-C differences between diagnostic groups and self-informant correlations and differences within diagnostic groups (alpha = 0.05, power = 0.80).^37^

## Results

### Clinical and demographic characteristics of the study sample

The clinical and demographic characteristics of the study sample are shown in Table 1. MCI participants were older than CN (*p* = 0.028) or SCD (*p* < 0.001), but there were no statistically significant differences between diagnostic groups in education or sex distribution. As expected, the MCI group performed worse than CN or SCD in all neuropsychological tests and there was no significant difference between SCD and CN. Table 2 shows the informant and self-rated MBI-C scores. Of note, both abnormal thought and perception and social inappropriateness were rarely endorsed, with 84% and 88% of patients, respectively, scoring zero in these domains, and 86% of informants reporting zero scores in both of these domains (Supplemental Table 3).

**Table 2.** Neuropsychiatric characteristics of the participants – MBI-C informant and self-rating.

MBI-C domain	Informant				Self-rating			
	CN (*M* ± *SD*)	SCD (*M* ± *SD*)	MCI (*M* ± *SD*)	Group differences	CN (*M* ± *SD*)	SCD (*M* ± *SD*)	MCI (*M* ± *SD*)	Group differences
Total score (0−102)	0.73 ± 1.61	3.95 ± 5.62	7.35 ± 9.53	CN < SCD** < MCI*; CN < MCI***	1.00 ± 1.74	4.79 ± 5.72	5.94 ± 6.63	CN < SCD**; CN < MCI***
Decreased motivation (0−18)	0.04 ± 0.20	0.55 ± 1.22	1.57 ± 1.22	CN < SCD* < MCI*; CN < MCI***	0.12 ± 0.43	1.00 ± 1.85	1.65 ± 2.60	CN < SCD*; CN < MCI***
Affective dysregulation (0−18)	0.12 ± 0.33	1.58 ± 2.99	2.55 ± 3.42	CN < SCD**; CN < MCI***	0.58 ± 1.17	2.13 ± 2.79	2.24 ± 2.68	CN < SCD**; CN < MCI***
Impulse dyscontrol (0−36)	0.58 ± 1.33	1.45 ± 2.10	2.52 ± 3.70	CN < MCI***	0.27 ± 0.60	1.24 ± 1.65	1.63 ± 2.25	CN < SCD*; CN < MCI***
Social inappropriateness (0−15)	0.00 ± 0.00	0.21 ± 0.53	0.35 ± 0.97	–	0.04 ± 0.20	0.26 ± 0.72	0.20 ± 0.61	–
Abnormal perception and thought content (0−15)	0.00 ± 0.00	0.16 ± 0.44	0.36 ± 1.05	CN < MCI*	0.00 ± 0.00	0.16 ± 0.37	0.23 ± 0.47	CN < MCI*

Difference between groups: Kruskal-Wallis with Dwass-Steel-Critchlow-Fligner post hoc test. MBI-C: Mild Behavioral Impairment–Checklist; CN: cognitively normal; SCD: subjective cognitive decline; MCI: mild cognitive impairment.

*p < 0.05; **p < 0.01; ***p < 0.001.

The MCI group scored higher than the CN group on the MBI-C total and domain scores in both informant and self-ratings, except for social inappropriateness (abnormal perception and thought content domain according to informant *p* = 0.049, according to patient *p* = 0.031, the rest was *p* < 0.001). SCD participants had higher MBI-C scores than CN in informant- and self-rated total scores (*p* = 0.006 and *p* = 0.002, respectively), decreased motivation (informant *p* = 0.041, patient *p* = 0.016) and affective dysregulation (informant *p* = 0.009, patient *p* = 0.006) and self-rated impulse dyscontrol (*p* = 0.010). The MCI group had higher MBI-C scores than SCD group in informant-rated total score (*p* = 0.038) and decreased motivation (*p* = 0.047), but there were no differences between these groups when rated by participants.

As for the informants, most of them were spouses (94 informants; 61.4%), fewer were descendants (50 informants, 32.7%) and the remainder (6 informants; 3.9%) were others (e.g., siblings, grandchildren).

### Self-informant rating discrepancies

Overall, self- and informant-rated MBI-C scores correlated weakly to moderately (*r*_s_ = 0.23–0.55, *p*s < 0.005; Table 3). No statistically significant differences between the self-informant correlations were observed when comparing the diagnostic groups (Supplemental Table 1). When comparing informant and self-rated scores of the same domain within diagnostic groups (Figure 1), self-rated scores were higher than informant-rated scores for affective dysregulation (*p* = 0.049) for the CN group and informant-rated scores were higher than self-rated scores in impulse dyscontrol for MCI group (*p* = 0.013).

**Figure 1.**
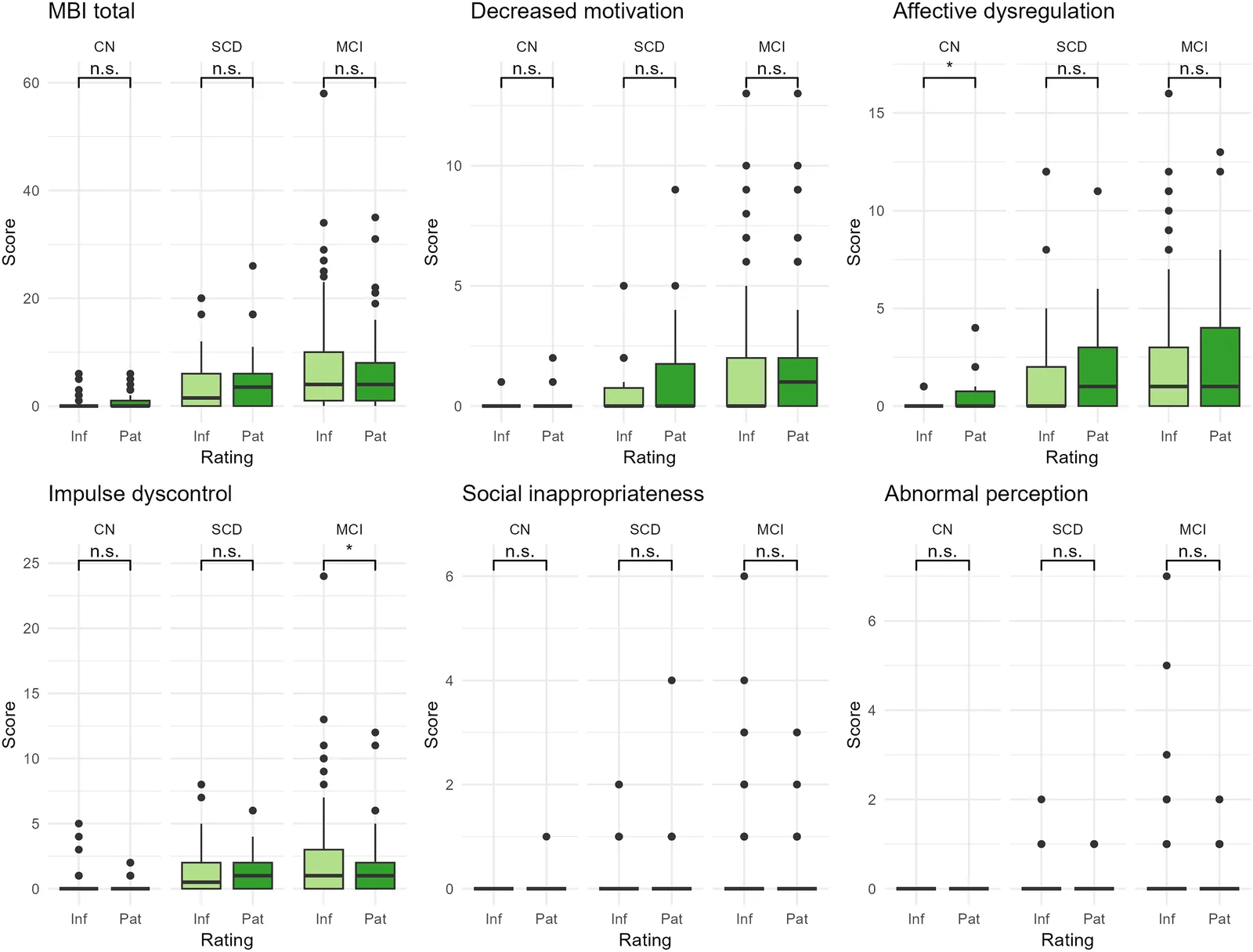
Comparison of MBI-C informant and participant scores within diagnostic groups. Self-rated scores were higher than informant-rated scores for affective dysregulation (*p* < 0.05) for the CN group and informant-rated scores were higher than self-rated scores in impulse dyscontrol for MCI group (*p* < 0.05). Comparisons were conducted with Wilcoxon signed rank-test for paired data. Difference between informant- and self-rated scores in CN Abnormal thought and perception domain could not be analyzed due to all scores being zero or very close to zero in both ratings. CN: cognitively normal; Inf: informant; MBI-C: Mild Behavioral Impairment–Checklist; MCI: mild cognitive impairment; n.s.: not significant; Pat: patient; SCD: subjective cognitive decline. *p < 0.05.

**Table 3.** Correlations between informant- and self-rated MBI-C scores.

	*r*_s_ (95% CI)	*p*
Total score	0.55 (0.41–0.68)	<0.001
Decreased motivation	0.52 (0.37–0.64)	<0.001
Affective dysregulation	0.49 (0.35–0.63)	<0.001
Impulse dyscontrol	0.53 (0.39–0.65)	<0.001
Social inappropriateness	0.23 (0.01–0.46)	0.004
Abnormal perception and thought content	0.41 (0.19–0.61)	<0.001

MBI-C: Mild Behavioral Impairment–Checklist; rs: Spearman correlation.

### Associations with cognitive domains

The results comparing linear regression analyses with informant- and self-rated scores as separate terms versus informant-self difference scores are presented in Supplemental Table 2. In summary, the models with separate scores (mean *R*^2^ = 0.06) had on average 0.04 higher *R*^2^ than difference scores (mean *R*^2^ = 0.02). In separate term models, informant scores were statistically significant in 10 (27.8%) models whereas participant scores were statistically significant in only 1 (2.8%) model after adjusting for multiple comparisons. Of all 36 difference score models, only impulse dyscontrol predicting language functions was significant after adjusting for multiple comparisons. Across all models, whenever the difference score was significantly related to cognition before p-value adjustment, significance was observed solely for the informant score (and not for the self-rated score) in the separate term model. No model showed both informant- and self-rated scores to be statistically significantly associated with cognition after adjusting for multiple comparisons; therefore, higher-order models were not pursued in this study.

Having shown that using separate MBI-C informant and participant scores increase *R*^2^ compared to difference scores, we finally analyzed the associations of MBI-C participant and informant scores and cognitive domains after adjusting for age, sex and education (Table 4). For informant-rated MBI-C measures, statistically significant associations that survived adjustment for multiple comparison were found between MBI-C total score and memory performance (β = −0.28, *SE* = 0.07, *p* = 0.002), decreased motivation and memory (β = −0.25, *SE* = 0.11, *p* = 0.003), affective dysregulation and global cognition (β = −0.29, *SE* = 0.07, *p* = 0.001), affective dysregulation and executive functions (β = −0.27, *SE* = 0.10, *p* = 0.004), affective dysregulation and memory (β = −0.31, *SE* = 0.10, *p* = 0.001). For self-rated MBI-C scores, an association was found only between decreased motivation and attention (β = −0.29, *SE* = 0.09, *p* = 0.001).

**Table 4.** Associations between MBI-C informant and patient scores after adjusting for covariates.

	Memory		Attention		EF		Language		Visuospat		Global	
	β	SE	β	SE	β	SE	β	SE	β	SE	β	SE
	MBI-C total											
MBI-C Informant	**−0** **.** 28*	−0.07	−0.12	0.06	−0.26	0.07	−0.24	0.07	−0.03	0.09	−0.24	0.05
MBI-C Patient	0.00	0.08	−0.14	0.07	0.00	0.08	0.01	0.08	0.07	0.10	−0.00	0.06
R^2^	0.28		0.23		0.26		0.31		0.07		0.33	
AIC	305.00		274.10		300.19		287.17		364.77		220.03	
	Decreased Motivation											
MBI-C Informant	**−0**.25*	0.11	−0.04	0.09	−0.21	0.10	−0.17	0.10	−0.01	0.14	−0.18	0.07
MBI-C Patient	−0.12	0.11	**−0**.29*	0.09	−0.17	0.10	−0.16	0.10	−0.13	0.14	−0.22	0.07
R^2^	0.31		0.27		0.30		0.33		0.08		0.38	
AIC	300.07		266.31		294.47		283.51		363.17		209.65	
	Affective Dysregulation											
MBI-C Informant	**−0**.31**	0.10	−0.13	0.09	**−0**.27*	0.10	−0.25	0.09	−0.16	0.12	**−0**.29**	0.07
MBI-C Patient	0.04	0.10	−0.12	0.09	0.00	0.10	−0.02	0.09	0.13	0.13	0.02	0.07
R^2^	0.29		0.23		0.27		0.32		0.08		0.35	
AIC	303.65		274.09		299.44		285.52		362.49		216.00	
	Impulse Dyscontrol											
MBI-C Informant	−0.21	0.10	−0.09	0.09	−0.24	0.10	−0.25	0.09	0.03	0.13	−0.19	0.07
MBI-C Patient	0.05	0.12	−0.10	0.11	0.14	0.12	0.17	0.11	0.10	0.15	0.10	0.09
R^2^	0.24		0.20		0.24		0.30		0.08		0.30	
AIC	311.92		277.79		303.63		288.38		363.45		225.62	
	Social Inappropriateness											
MBI-C Informant	−0.01	0.20	−0.06	0.18	−0.04	0.20	−0.10	0.19	−0.01	0.24	−0.05	0.14
MBI-C Patient	0.07	0.24	−0.04	0.21	0.07	0.24	0.06	0.22	0.11	0.29	0.08	0.17
R^2^	0.21		0.19		0.20		0.26		0.07		0.28	
AIC	316.85		280.37		309.99		295.45		363.81		229.57	
	Abnormal Thoughts and Perception											
MBI-C Informant	−0.06	0.21	−0.06	0.18	−0.16	0.20	−0.07	0.20	0.07	0.26	−0.07	0.15
MBI-C Patient	−0.10	0.28	−0.14	0.24	−0.03	0.27	−0.03	0.26	−0.04	0.34	−0.08	0.20
R^2^	0.23		0.21		0.23		0.26		0.07		0.29	
AIC	314.64		276.62		305.91		295.57		364.85		227.51	

p-values adjusted using Holm correction considering analyses using MBI domains as families. Multiple comparison adjustment did not include the covariates of sex, age and education. MBI-C: Mild Behavioral Impairment–Checklist; EF: Executive functions, Visuospat: Visuospatial functions.

*p < 0.05; **p < 0.01; ***p < 0.001 in multiple-comparison adjusted p-values.

## Discussion

This is the first clinical study using both informant- and self-rated versions of the MBI-C in a neuropsychologically characterized memory clinic sample of non-demented older adults. We compared self and informant ratings of 127 individuals across cognitive continuum preceding dementia and examined their associations with the patients’ cognition. Both self and informant ratings were higher in SCD and MCI compared to the control group. Importantly, informant-rated NPS severity in the clinical (SCD + MCI) group was associated with impairment across multiple cognitive domains, contrasting with the lack of substantial correlation with the self-rated NPS. On average, informants reported more severe impulse dyscontrol than participants themselves. Overall, patient-informant agreement on NPS was weak-to-moderate.

### NPS across the cognitive continuum

Our study builds upon previous population-based studies by examining NPS across the cognitive continuum preceding dementia.^24,25^ Consistent with these studies, we found that both participants and informants most frequently reported decreased motivation, affective dysregulation and impulse dyscontrol, while social inappropriateness and abnormal perception and thought content were rarely reported. As expected, NPS were higher in both clinical groups than in controls.

We observed continuing gradient of worsening informant-rated NPS through the cognitive continuum (CN-SCD-MCI) in the total score and in the decreased motivation domain. Informants also reported higher affective dysregulation in both SCD and MCI compared to CN, with no difference between clinical groups. Our results are consistent with previous clinical studies showing increasing NPS severity across the cognitive continuum as measured by the MBI-C or the NPI-Q.^2,4,38^ Higher impulse dyscontrol and abnormal perception and thought content emerged only in MCI compared to CN, in line with studies showing time course of various NPS.^2,39^

Although patient administration of the MBI-C has begun to be used more often in studies, our study is the first investigation using MBI-C self-report in clinical population.^21–25^ A recent community-based study using the self-rated MBI-C found that the MCI group reported more symptoms than CN in total score, decreased motivation, affective dysregulation and abnormal perception and thought content domains.^23^ Our study aligns with these results; in addition, we found higher scores in the impulse dyscontrol domain in MCI group compared to CN. On the other hand, Leow et al. did not find any difference between SCD and CN in any MBI-C domain contrasting with our study, where the SCD group had higher scores in the majority of MBI-C domains.^23^ This discrepancy likely reflects sample differences: clinical SCD cohorts generally have a higher prevalence of subjective cognitive complaints, NPS, and amyloid positivity than community samples, increasing the likelihood of detecting NPS differences.^10^^40–43^ Indeed, other clinical studies using domain-specific self-report questionnaires have also found higher NPS not only in MCI but also in SCD group compared to CN.^44–46^ In contrast, we observed no significant differences between SCD and MCI in any self-rated MBI-C domain, which aligns with another clinical study reporting comparable levels of self-rated depressive symptoms in these groups.^45^

Altogether, our results confirm the presence of mild NPS in both clinical groups of nondemented older adults. We observed that CN and SCD groups, although similar in cognitive performance, differed in several NPS as reported by themselves or their informants. Cognition-related anxiety and worry are among the main reasons for which SCD individuals seek medical help and may be overlapping with MBI, but our study shows that there are also other NPS, such as apathy and impulse dyscontrol, that the individuals experience and informants observe in this stage.^6^ Meanwhile, SCD and MCI groups, although cognitively different by definition, only differed in informant-rated total MBI-C and decreased motivation scores, suggesting that increasing cognitive impairment is associated with greater NPS severity only in certain domains. This pattern partially aligns with a finding that Aβ-positive SCD individuals did not differ from Aβ-positive MCI in any NPI-Q score.^47^ The slightly higher NPS levels of apathy observed in our study (compared to studies using NPI-Q) may indicate a higher sensitivity of the MBI-C for capturing these changes in nondemented older adults.

### Self-informant rating discrepancies

Analogously to previous studies, informant and self MBI-C ratings showed only weak-to-moderate correlations.^24,25^ We also found that informants reported systematically more impulse dyscontrol symptoms than patients with MCI, consistent with previous research highlighting the greatest self-informant discrepancy in this domain.^25^ Patients may underreport behaviors like irritability, impulsivity, or aggression—either due to lack of insight or an attempt to maintain a positive self-image—whereas informants may overestimate the severity of these symptoms based on their observations, because it occurs often in social situations and is associated with perceived burden.^48–50^ In a previous study, items with the greatest self–informant discrepancy were primarily related to negative personality traits, supporting this interpretation.^25^

We observed no significant self-informant discrepancy in the affective dysregulation domain. Prior studies on depressive and anxiety symptoms have yielded mixed results, with some finding higher self-ratings and others higher informant ratings.^16,24,51,52^ Only two population studies examined self-informant differences in NPS with MBI-C: one found no self-informant difference in affective symptoms, whereas the other reported that participants endorsed more affective symptoms than their informants, which we did not confirm in our clinical groups.^24,25^ Notably, the raw MBI-C scores (including affective dysregulation) were higher in our clinical groups than in those community samples.^24^ In a clinical population, affective symptoms are typically more pronounced and thus more apparent to informants, whereas in community samples, the symptoms tend to be milder and more difficult for informants to detect.^10,42^ As a result, informants may not observe the milder symptoms that participants still experience. Another explanation of these differences between self- and informant-rated depressive symptoms could be their different nature (externally observable versus internally experienced).^53^

### Associations with cognitive domains

Our findings highlight that informant and patient NPS ratings have distinct relationships with objective cognition. While the overall variance in cognition explained by NPS ratings was modest (maximum 15% of a cognitive domain), informant-rated NPS showed consistently stronger links to cognitive performance than self-rated NPS. We found that higher informant total and domain scores were associated with poorer memory, executive, and global cognitive function, whereas self-ratings showed only one association (higher self-rated apathy with worse attention). This pattern aligns with previous reports: informant-rated MBI-C scores correlate with worse global cognition in clinical sample and with memory and executive deficits in community sample, whereas self-rated MBI-C shows minimal cognitive associations.^4,54,55^ However, the association of the self-rating with attention in our study was not found in informant ratings, highlighting a unique contribution of the patient self-reports in this instance. Of note, this association was as strong as those found between informant ratings and other cognitive domains, suggesting that patient self-ratings can still provide valuable insight.

Discrepancies between patient and informant NPS ratings have been observed to widen with more severe impairment in memory and executive function, likely reflecting emerging anosognosia (unawareness of deficits) in the patient.^15,18^ Anosognosia is linked to memory impairment and can manifest even in prodromal stages of neurodegenerative diseases.^19^^56–58^ Notably, in our analyses the informant rating alone was a better predictor of cognitive outcomes than the informant-patient difference score, suggesting that the informant perspective largely drives the association between NPS and cognitive impairment.

These discrepancies between patient and informant reports also raise the question of whether clinician-administered assessments could provide added value. The MBI-C administration instructions also allow for a clinical interview. Although we did not examine this mode of administration, studies with related instruments suggest potential benefits: for instance, the clinician-rated Apathy Evaluation Scale (AES-C) predicted progression from MCI to dementia better than self- or informant-reports.^16^ Also, the Neuropsychiatric Inventory clinician version (NPI-C) was developed to enhance objectivity through clinical judgment.^59^ Similar advantages may apply to the MBI-C, but further validation of clinician interview administration is needed.

### Limitations

This study has several limitations. First, the sample came from a memory clinic, which limits the generalizability to the general population—clinical populations tend to exhibit a higher prevalence of NPS than community samples.^10,42^ Second, we examined an etiologically heterogeneous sample without biomarkers. However, in this way our sample reflects clinical practice where biomarkers are often missing. Third, our informant-based measures did not account for informant characteristics. Recent research indicates informant factors (such as age, gender, or relationship to the patient) can influence reported NPS.^60^ Future studies should consider adjusting for informant characteristics. Fourth, domains of social inappropriateness and abnormal perception and thought content were very infrequent in our sample, as expected in early-stage AD, which may cause small differences and lack of association with cognition in those domains.^1^ Analyses using these domains did not unequivocally meet the assumptions of linear regression and might benefit from different statistical approaches than the ones employed here. However, for most models, the assumptions of linear regression were met. Finally, the correlations between self- and informant reports would ideally be interpreted with the reliability of these measures in mind. However, to estimate reliability in cross-sectional data informatively would require establishing the dimensionality of the MBI-C with factor analysis, which our sample size is not equipped to do. Therefore, it is a limitation of our study that we have used the MBI-C scales with the *a priori* structure rather than testing whether this structure holds in factor analysis and provides reasonable reliability estimates.

### Conclusion

This clinical study highlights important self-informant discrepancies in reporting NPS in older adults without dementia. Informant ratings consistently showed stronger associations with cognitive deficits particularly in memory and executive function suggesting they may offer a more accurate reflection of the patient's current cognitive status. Furthermore, impulse dyscontrol was consistently underestimated by patients. Yet, self-ratings provided relevant data, with self-perceived apathy correlating with attention deficit, highlighting a patient perspective that informants may miss. Our findings have important clinical implications supporting the need for complementing self-reports with information from their informants even in early stages of neurodegenerative diseases. Future studies should examine these discrepancies on biomarker-defined cohorts and from longitudinal perspectives.

## Supplemental Material

sj-docx-1-alz-10.1177_13872877251401620 - Supplemental material for Comparing self and informant perspectives on neuropsychiatric symptoms in older adults in a memory clinic Supplemental material, sj-docx-1-alz-10.1177_13872877251401620 for Comparing self and informant perspectives on neuropsychiatric symptoms in older adults in a memory clinic by Monika Krejčí, Veronika Matušková, Toni T Saari, Hana Horáková, Šárka Borovská, Jan Laczó, Jakub Hort and Martin Vyhnálek in Journal of Alzheimer's Disease

sj-xlsx-2-alz-10.1177_13872877251401620 - Supplemental material for Comparing self and informant perspectives on neuropsychiatric symptoms in older adults in a memory clinic Supplemental material, sj-xlsx-2-alz-10.1177_13872877251401620 for Comparing self and informant perspectives on neuropsychiatric symptoms in older adults in a memory clinic by Monika Krejčí, Veronika Matušková, Toni T Saari, Hana Horáková, Šárka Borovská, Jan Laczó, Jakub Hort and Martin Vyhnálek in Journal of Alzheimer's Disease

sj-xlsx-3-alz-10.1177_13872877251401620 - Supplemental material for Comparing self and informant perspectives on neuropsychiatric symptoms in older adults in a memory clinic Supplemental material, sj-xlsx-3-alz-10.1177_13872877251401620 for Comparing self and informant perspectives on neuropsychiatric symptoms in older adults in a memory clinic by Monika Krejčí, Veronika Matušková, Toni T Saari, Hana Horáková, Šárka Borovská, Jan Laczó, Jakub Hort and Martin Vyhnálek in Journal of Alzheimer's Disease
