# Supplementary material for: Comparing self and informant perspectives on neuropsychiatric symptoms in older adults in a memory clinic
Source: J Alzheimers Dis. 2025 Dec 15;109(2):1036–47. doi: 10.1177/13872877251401620 (PMC13687618; doi:10.1177/13872877251401620)
Supplement: Supplementary file 1 [file sj-docx-1-alz-10.1177_13872877251401620.docx]

**Supplemental Material**

**Comparing self and informant perspectives on neuropsychiatric symptoms in older adults in a memory clinic**

| **Supplementary Table 1.** Comparison of MBI-C informant-patient Spearman correlations between diagnostic groups | | | | | | | | | | |
| --- | --- | --- | --- | --- | --- | --- | --- | --- | --- | --- |
|  | *r*_s_ (95% CI) | | | |  | *r*_s_ difference (95% CI) | | | |  |
|  | CN | SCD | MCI |  | | | CN-SCD | SCD-MCI | CN-MCI | |
| Total score | 0.35 (-0.05-0.74) | 0.58 (0.32-0.8) | 0.45 (0.23-0.64) |  | | | -0.23 (-0.69-0.22) | 0.13 (-0.2-0.42) | -0.1 (-0.54-0.31) | |
| Decreased motivation | -0.06 | 0.38 (0.07-0.69) | 0.48 (0.29-0.64) |  | | | -0.44 | -0.1 (-0.47-0.26) | -0.54 | |
| Affective dysregulation | 0.34 | 0.42 (0.1-0.68) | 0.48 (0.28-0.66) |  | | | -0.08 | -0.06 (-0.42-0.26) | -0.14 | |
| Impulse dyscontrol | 0.44 (-0.1-0.84) | 0.57 (0.3-0.78) | 0.46 (0.28-0.63) |  | | | -0.13 (-0.73-0.34) | 0.11 (-0.22-0.39) | -0.02 (-0.58-0.43) | |
| Social inappropriateness | – | 0.2 (-0.18-0.61) | 0.25 (-0.04-0.51) |  | | | – | -0.04 (-0.52-0.46) | – | |
| Abnormal perception and thought content | – | 0.28 (-0.13-0.73) | 0.42 (0.16-0.65) |  | | | – | -0.15 (-0.64-0.34) | – | |
| Values are shown as Spearman correlations and raw differences with 95 % confidence intervals. Since some of the groups are small and data are zero-inflated, computation of correlations and confidence intervals was not feasible for all groups or comparisons. MBI-C: Mild Behavioral Impairment–Checklist; CN: cognitively normal; SCD: subjective cognitive decline; MCI: mild cognitive impairment. | | | | | | | | | | |

**Supplemental Table 2.** Comparison of MBI-C difference scores versus separate informant and patient scores for predicting cognitive domains. See Excel file.

**Supplemental Table 3.** Frequencies of zero responses for each MBI-C domain. See Excel file.

**Supplemental Figure 1.** MBI-C score distributions for SCD and MCI before transformation.

Abn: abnormal perception and thought content; aff: affective dysregulation; imp: impulse dyscontrol; int: decreased motivation; mbitot: MBI-C total score; _i: informant rating; _p: patient rating.

**Supplemental Figure 2.** Log(x+1) transformed MBI-C score distributions for MCI and SCD participants

Abn: abnormal perception and thought content; aff: affective dysregulation; imp: impulse dyscontrol; int: decreased motivation; mbitot: MBI-C total score; _i: informant rating; _p: patient rating; lg: logarithm transformation(x+1).
